# Microgel with a Core—Shell Particulate Structure Formed via Spinodal Decomposition of a Diblock Ionomer Containing a Doped Hydrophobic Moiety

**DOI:** 10.3390/gels11040231

**Published:** 2025-03-22

**Authors:** David Julius, Jim Yang Lee, Liang Hong

**Affiliations:** Department of Chemical and Biomolecular Engineering, National University of Singapore, 4 Engineering Drive 4, Singapore 117585, Singapore; david_julius@hotmail.com (D.J.); cheleejy@nus.edu.sg (J.Y.L.)

**Keywords:** diblock-ionomer, microgel, colloidal dispersion, spinodal decomposition, ATRP synthesis, block copolymer, amphiphilic property

## Abstract

This study explored the formation of soft colloidal particles from a diblock ionomer (DI) with the monomeric composition (acrylonitrile)_x_-co-(glycidyl methacrylate)_y_-b-(3-sulfopropyl methacrylate potassium)_z_—abbreviated as (A_x_G_y_)S_z_, where x >> z > y. A colloidal dispersion was generated by introducing water into the pre-prepared DMSO solutions of DI, which led to micelle formation and subsequent coagulation. The assembly of the hydrophobic (A_x_G_y_) blocks was influenced by water content and chain conformational flexibility (the ability to adopt various forms of conformation). The resulting microgel structure (in particle form) consists of coagulated micelles characterized by discrete internal hydrophobic gel domains and continuous external hydrophilic gel layers. Characterization methods included light scattering, zeta potential analysis, and particle size distribution measurements. In contrast, the copolymer (A_x_G_y_) chains form random coil aggregates in DMSO–H_2_O mixtures, displaying a chain packing state distinct from the hydrophobic gel domains as aforementioned. Additionally, the amphiphilic glycidyl methacrylate (G) units within the (A_x_G_y_) block were found to modulate the microgel dimensions. Notably, the nanoscale hydrogel corona exhibits high accessibility to reactive species in aqueous media. The typical microgel has a spherical shape with a diameter ranging from 50 to 120 nm. It exhibits a zeta potential of −65 mV in a neutral aqueous medium; however, it may precipitate if the metastable colloidal dispersion state cannot be maintained. Its properties could be tailored through adjusting the internal chain conformation, highlighting its potential for diverse applications.

## 1. Introduction

A hydrogel is structurally a hydrophilic polymer network possessing adequate softness, toughness, and stretchability. In many cases, it also exhibits biocompatibility and biodegradability after being swelled with water. This is because each monomer unit of the polymer network carries pendant hydrophilic functional group(s), which can be cationic -NR_3_^+^·Cl^−^, anionic -SO_3_^−^Na^+^, or nonionic (-CH_2_CH_2_O-) type, and the hydrogel often possesses super porous structure [1,2]. To date, hydrogel research has primarily focused on constructing 3D hydrophilic networks tailored for specific biomedical applications, including cell cultures, drug delivery, wound dressing, and tissue engineering [3,4]. Regarding the techniques employed to construct hydrogel networks, chemical crosslinking employs conventional [5] and reversible [6] covalent bonds to deploy crosslinking points. In contrast, physical crosslinking typically involves *i*. ionic interactions; *ii*. hydrophobic associations of water-soluble polymer main chains with hydrophobic end groups, side chains, and monomer units; *iii*. thermally induced phase transitions of amphiphilic block copolymers at either the lower or upper critical solution temperature (LCST or UCST), which results from the aggregation of hydrophobic blocks surrounded by hydrophilic loops, namely self-assembly, accompanying collapse of polymer chains. UCST happens more often than LCST because endothermicity normally assists with the solvation of polymer chains; *iv*. hydrogen bonding between polymer chains; *v*. Formation of a racemic crystallite due to stereo-complexation between the side-chain groups that are *d* and *l* enantiomers; and *vi*. Lewis base-acid type associations, e.g., donor-acceptor interactions [7]. It is logical to infer that both physically and chemically crosslinked hydrogels, generated from homogeneous solutions, produce continuous 3D networks with the dimensions far beyond the nanoscale. This is because these crosslinking systems ensure that the gelation to takes place uniformly in the reaction vessels or molds. The concept of microgels, which often are hydrogels of colloidal sizes, has gained attention due to their surface-dominant characteristics. This surface dominance indicates a dynamic outward behavior resulting from the greater freedom of movement of polymer strands. As a result, microgels exhibit enhanced sensitivity to external stimuli such as temperature, pH, and solvent conditions [8,9]. This sensitivity can be attributed to a decrease in crosslink density from the center to the surface of individual hydrogel colloids. Consequently, hydrogel latex particles have various applications in the biomedical field [10].

There are several ways to prepare microgel colloids, typically through the following: *i.* polymerizing hydrophilic monomers in a colloidal suspension to construct a hybrid gel, where hydrophobic latex particles are embedded in the hydrogel formed [11]; *ii*. shrinkage of the less hydrophilic PHPMA block in an amphiphilic diblock copolymer, poly(mathacrylic acid)-*b*-poly(2-hydroxypropyl methacrylate), in water to form nanoparticles with a fully extended hydrogel corona [12]; *iii*. pH- and solvent-induced isolated clamping of a pertinent water-soluble polymer, such as gelatin [9]; *iv*. grafting hydrophilic oligomers to form water-insoluble nano or sub-micron seeds [13,14]; and *v.* microfluidic generation using different devices and various approaches (on/off-chip gelation) to form microgel particles [15]. It is worth noting that strategies *ii* and *iii* fundamentally rely on thermodynamically driven spinodal decomposition, during which certain polymer chains or segments undergo desolvation and aggregation [16].

To date, microgel colloids composed entirely or partially of hydrophilic polymers have garnered intensive attention because of their soft, responsive, dynamic, and biocompatible features [17]. Contemporarily, microgels, often referred to as hydrogel microrobots, have been explored for various biomedical applications, including drug delivery, minimally invasive surgery, tissue engineering, stem cell therapy, and chemical sensing [18,19]. Additionally, hydrogel microparticles have found commercial applications in water treatment, fuel production, and printing ink industries [20].

Hydrogel microparticles are primarily constructed by methacrylic acid, acrylamide, and ethylene glycol polymer chains, along with naturally occurring polymer ingredients due to their high water uptake and stimuli-responsive behaviors [21,22]. The conventional characterization techniques for characterizing microgel structures include atomic force microscopy (AFM), electron microscopy (SEM and TEM), and static/dynamic light scattering (S/DLS). Beyond these methods, advanced approaches such as super-resolution microscopy have further enhanced the structural analysis capabilities of microgel [23].

**Figure 1:**
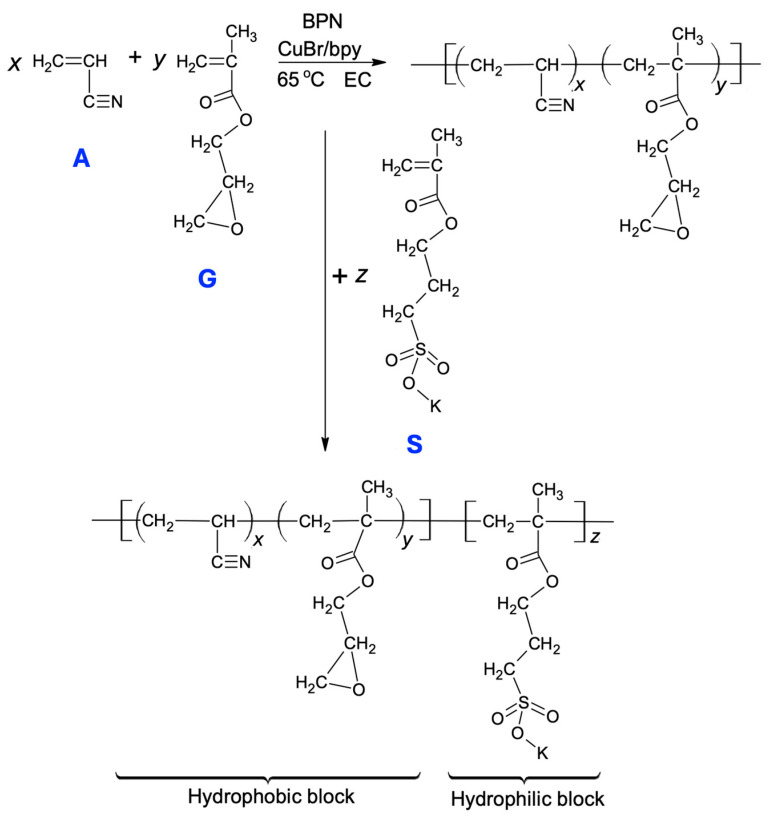
A schematic illustration for the ATRP synthesis of the diblock ionomer, (A_x_G_y_)S_z_.

In this study, we synthesized an asymmetric DI, (A_x_G_y_)S_z_, in which (x + y >> z), using ATRP, a living free-radical polymerization approach. This synthesis can be depicted in Figure 1. With the exception of ATRP, the other two parallel methods of controlled radical polymerization are reversible addition, fragmentation chain-transfer (RAFT) and nitroxide-mediated polymerization (NMP). ATRP uses a very low molar ratio of the activator, LnCuX (e.g., bpy·CuBr), to monomers; however, the removal of LnCuX_2_ from the polymer synthesis system remains an area for improvement. The drawback of RAFT is that the *thiocarbonylthio* compounds, which serve as the chain transfer reagent, can impart odor and color to the final polymer [24]. Furthermore, NMP is inappropriate for polymerizing methacrylate monomers due to side reactions and/or slow recombination of the polymer radical with nitroxide [25].

The originality of this work lies in the design of the block copolymer structure, where the hydrophobic block is infused with the amphiphilic unit G. This amphiphilic doping leads to several unusual colloidal behaviors, which are highlighted in the final paragraph below. A key challenge in synthesizing this type of DI is selecting a solvent that accommodates both blocks with opposing polarities and does not affect the advancment of polymerization [26]. The combination of a low-volatility aprotic solvent with water helps to maintain the amphiphilic polymer chains soluble; the water component may cause disproportionation of the Cu(I)-based catalyst and deactivation of ATRP [27]. In the present preparation, dimethyl sulfoxide (DMSO, CH_3_SOCH_3_) was added to the polymerization system for the first block, using ethylene carbonate (EC) as the solvent, to facilitate the extension of the S_z_ block onto the (A_x_G_y_) block. This binary solvent ensures that the polymerization system remains in a homogeneous liquid state throughout the second growth stage, through which the formation of the diblock copolymer is finalized. Furthermore, with regard to the (A_x_G_y_) block, it is well known that polyacrylonitrile is intrinsically hydrophobic, as indicated in a previous study [28]. This hydrophobicity primarily arises from its very high cohesive energy density (solubility parameter, δ = 26.1 MPa^0.5^ at 25 °C), despite the potential for its nitrile groups to be converted to super-hydrophilic COONa and CONH_2_ groups [29]. Although poly(glycidyl methacrylate) is inherently amphiphilic [30], the lower y/x ratio makes the (A_x_G_y_) block predominantly hydrophobic.

Consequently, when water is introduced into the DMSO solution of a DI obtained from the ATRP system, the hydrophobic (A_x_G_y_) blocks of the DI progressively aggregate at ambient temperature. Thermodynamically, this process evolves from an initial single-phase region to a binodal region and then transitions to a spinodal region [31]. As a result of this phase transition, microgel colloids form with a hydrophobic (A_x_G_y_) core surrounded by a hydrophilic corona composed of S_z_ blocks, termed as a primary colloidal or microgel particle, resembling an oligomer-derived micelle structure. The G units distributed within the (A_x_G_y_) blocks function as pivot points, enabling the chain rotations of acrylonitrile (A) segments between G units to achieve greater conformational flexibility compared to the homo-A_x_-block. This flexibility affects the micelle structure formed during self-assembly and then the microparticles consisting of the micelles in the DMSO-H_2_O medium. To experimentally investigate the proposed structural effects, various instrumental techniques were utilized, including light scattering intensity, zeta potential measurements, and dynamic light scattering particle size distribution analysis. In addition to analyzing the colloidal dispersions, transmission electron microscopy was used to observe the morphologies of colloidal particles separated from the dispersions where spinodal decomposition has taken place. Notably, the study found that the z/(x + y) ratio influences the structure of colloidal particles formed during spinodal decomposition. The highest ratio, 10/(50 + 4), leads to the formation of spherical colloidal particles, while the lower ratios result in more extensive aggregation of micelles. This suggests that greater conformational flexibility of the hydrophobic (A_x_G_y_) block, associated with lower y/x ratios, 10/(100 + 4) and 10/(150 + 4), leads to loosely constructed colloidal particles with lower surface charge extents, which allow for further coagulation of the primary colloidal particles. These findings provide valuable insights for designing microgel particles with a hydrophobic core and a hydrophilic outer layer using (A_x_G_y_)S_z_ and similar analogs with chemically tunable hydrophobic block structures. In comparison to this study, a previous publication indicated that the varying softness of the hydrophobic block’s chain can alter the rigid-flexible properties of the resulting colloidal particles [32]. However, that study did not address the concept of monomer doping presented in the current work. In summary, this study introduces the idea of adjustable hydrophobic chain packing density inside individual microgel particles with a hydrophilic corona on each by incorporating dilute amphiphilic units into the hydrophobic blocks of the diblock copolymer.

## 2. Results and Discussion

### 2.1. Monitoring the Chain Growth of (A_x_G_y_)S_z_ in One-Pot ATRP System

As the first step of ATRP, the growth of the (A_x_G_y_) block took place in EC solvent as it is a good solvent for this polymer block. Compared to the other aprotic solvents, such as propylene carbonate (PC), dimethyl formamide (DMF), and DMSO, the polymerization rate is higher in ethylene carbonate (EC) [33]. However, the ionic monomer S is insoluble in EC, making it impossible to grow the S_z_ block on the (A_x_G_y_) block using EC as the sole solvent. We selected DMSO as the co-solvent to dissolve S because DMSO has a higher dipole moment (3.96 D) compared to the commonly used polar aprotic solvents DMF (3.82 D) and DMAc (3.72 D). Consequently, DMSO can more effectively coordinate with the K^+^ ion of each S monomer molecule, allowing for faster dissolution of the S monomer. Several previous publications applied ATRP to synthesize acrylic-based block ionomers in polar aprotic solvents, such as DMSO or DMF, with water as a co-solvent to address the solubility issues of ionic monomers [26,34]. However, the presence of water in the polymerization medium limits the growth of the hydrophobic block, leading to relatively short hydrophobic segments and low molecular weights. Therefore, a water-free solvent system is essential for achieving sufficiently long hydrophobic blocks before extending the hydrophilic blocks.

This section provides a detailed account of our ATRP synthesis, covering feed composition, reaction temperature, and polymerization time. During the preparation of the (A_x_G_y_) block, we fixed the feed compositions with the three molar ratios surrounding the typical ratio G/A = 0.04, identified as optimal for the self-assembly of (A_x_G_y_)S_z_ [35]. It is well established that the hydrophilic–lipophilic balance (HLB) of a random copolymer, comprising both hydrophobic and hydrophilic monomers, significantly influences its self-assembly behavior and the resulting micelle morphology in an aqueous medium [36]. It is rational that the G_y_ units in the (A_x_G_y_) blocks serve as spatial pivots, reducing the packing density of A_x_ segments. Specifically, the G_y_ units weaken the association of A_x_ segments due to a notable gap in the thermodynamic solubility parameters between polyacrylonitrile and poly(glycidyl methacrylate) [37,38]. Consequently, the aggregation of the hydrophobic block of the DI is not overwhelming through the course of forming microgel particles. This point will be further elaborated in the following sections.

**Figure 2:**
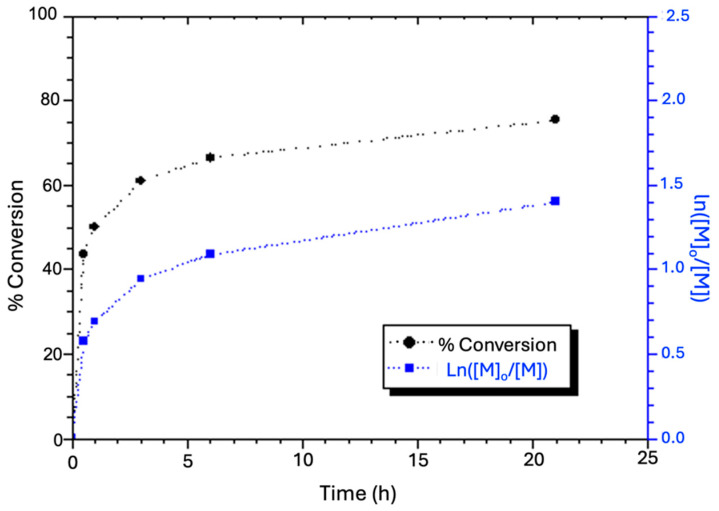
The ATRP synthesis time course vs. monomer conversion (●, refer to Section 4.3.1.a) and the logarithm of monomer consumption (■) in the ATRP synthesis of the A_x_G_y_ block at 65 °C in EC solvent.

**Figure 3:**
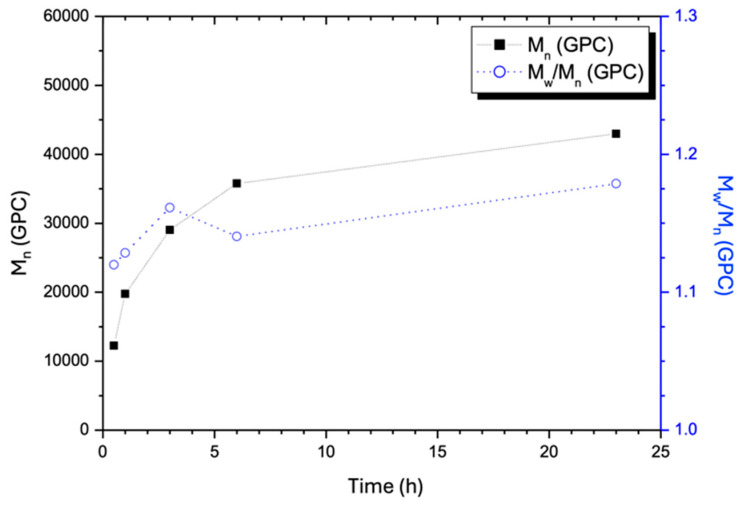
Average molecular weight, ${\bar{M}}_{n}$ (■) and the dispersity index, PDI (○) of (A_x_G_y_) synthesized at 65 °C in EC via ATRP as a function of the polymerization time using the feed as indicated previously.

Although the present synthesis included a series of feed compositions, the feed with the molar ratio of [A]_0_/[G]_0_/[RX]_0_/[CuX]_0_/[bpy]_0_ = 100/4/1/0.1/0.3 was chosen to examine the monomer conversion and polymer formation with time, from which the results are shown in Figure 2 and Figure 3. In the feed, RX (2-bromopropionitrile) serves as the initiator, CuX is Cu^I^Br as the polymerization catalyst, and bpy (2,2′-bipyridyl) acts as the catalyst ligand. The rest of the feeds maintained the same molar ratio of monomers (A + G) to the catalyst (CuX), as well as the consistent ratios of other components to the catalyst. As a result, the copolymers A_x_G_y_ must have comparable molar masses, as did the copolymers (A_x_G_y_)S_z_, provided that the reaction conditions, including duration, concentration, and temperature, remained unchanged because this inference is compliant with the ATRP mechanism. Figure 2 demonstrates that monomer conversion increased over time, reaching 63% in approximately 6 h, after which it gradually leveled off. Visually, the reaction mixture changed from an initial dark red solution to green after about 2 h, indicating significant progress in polymerization, consistent with the previous reports [33]. Hence, the reaction time of 6 h is considered optimal as it marks the turning point of the monomer conversion trend. It is thus termed as the optimal conversion time. The logarithmic plot indicates a non-first-order kinetics, as the reaction rate depends on the polymer chain with a transferable halogen atom and the catalyst, consistent with previous conclusions [39].

Figure 3 illustrates the changes in average molecular weights (${\bar{M}}_{n}$ and ${\bar{M}}_{w}$) and polydispersity index (PDI = ${\bar{M}}_{w}/{\bar{M}}_{n}$) for the hydrophobic block A_x_G_y_ as a function of the polymerization time. As expected, molecular weights increased with time, while polydispersity remained consistently in the range between 1.1 and 1.2 throughout the polymerization course, indicating that the conditions ensured the mechanism of ‘living polymerization’. The slope of the plot of ${\bar{M}}_{n}$ vs. polymerization time became lower after the optimal conversion time. At this optimal point, ${\bar{M}}_{n}$ reaches 36,000 Da, approximately each chain has a composition of A_637_G_26_ and its PDI slightly decreases to 1.15. Beyond this conversion time, the PDI increases gradually, indicating that as ${\bar{M}}_{n}$ increases further, chain growth becomes less uniform due to restricted mass diffusion caused by higher viscosity. A narrower A_x_G_y_ chain length distribution, namely ${\bar{M}}_{w}/{\bar{M}}_{n}$ is close to one, is preferred, as it would bring about more uniform hydrophobic nucleation sites in the binodal state, happening in the DMSO-water medium.

As previously described, the ATRP synthesis was followed by introducing a DMSO solution of ionic monomer S into the ATRP system to initiate the growth of the hydrophilic S_z_ block on the A_x_G_y_ chains. Due to the lack of a suitable solvent allowing optimal performance of the GPC column to dissolve the (A_x_G_y_)S_z_ species for the measurement of molecular weight throughout the polymerization, there is a lack of GPC analysis results for (A_x_G_y_)S_z_. Therefore, a 20-h reaction period was chosen to ensure a monomer conversion to attain S_z_ of approximately 65–75%, as estimated according to Figure 2. Thus, the polymerization degree of S_z_ can be approximated to fall between 98 and 113, based on the A_637_G_26_ chain count, the indicated monomer conversion range described above, and the amount of monomer S added to this polymerization system initially. Therefore, the projected DI chain composition lies between (A_637_G_26_)S_98_ and (A_637_G_26_)S_113_, with an estimated molecular weight of the DI ranging from 61,500 to 65,200 Da. This reasoning assumes that the three monomers incorporate into the Cu(I)-mediated free radical chains with identical kinetics. As a result, extending the reaction time (to 20 h) favored the growth of the S_z_ block; the 65–75% conversion range should hence lead to a greater ratio of S to A monomer units in either (A_637_G_26_)S_98_ or (A_637_G_26_)S_113_ compared to the monomer ratio of S to A in the feed. Additionally, the molar ratio of S to A plus G in the synthesized DI must be controlled within a specific limit in order to maintain homogeneity in the polymerization system when the S_z_ block grows in the binary EC-DMSO solvent (*v*/*v* ≈ 1). If a longer S_z_ block was attempted, the resulting DI chains would become insoluble in the polymerization solvent. Lastly, it should be reiterated that in the following paragraphs, samples are labeled by using the molar ratios of the ATRP feed compositions, such as (A_50_G_4_)S_10_, for simplicity. It is understood *that these labels do not reflect the actual polymer chain compositions*.

### 2.2. The DI Chain Structures and Their Colloidal Dispersion States in Water Deduced from Zeta Potential Measurement

**Figure 4:**
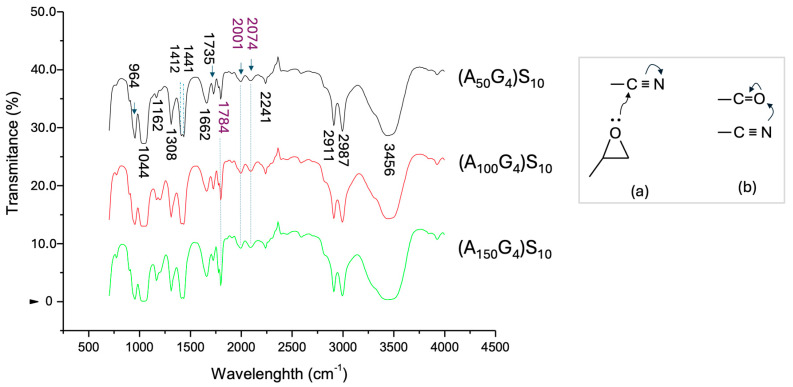
The FT-IR spectra of the three DI samples, which are labeled using the sample names specified by the monomer feeds, i.e., (A_50_G_4_)S_10_, (A_100_G_4_)S_10_, and (A_150_G_4_)S_10_. Insert: (**a**,**b**) show charge induction effect on the electron density in C≡N triple bond (**a**) and in C=O double bond (**b**). The corresponding wavenumber readings are labeled in purple, and the absorption bands are connected by dash lines.

The FT-IR spectra of the three DI samples (Figure 4), obtained as described in Section 4.2, display the characteristic fingerprints of the expected organic functional groups. (1) The spectra reveal a small amount of hydrate molecules associated with the ionic -SO_3_K groups, indicated by the characteristic absorption peaks at 3456 and 1662 cm^−1^. Additionally, all three monomer units contribute to the aliphatic C-H stretching in the range of 2911 to 2987 cm^−1^. (2) The nitrile group of the A_x_ block is identified by the C≡N stretching frequency at 2241 cm^−1^. Two additional absorption bands are observed at lower frequencies compared to the standard C≡N stretching, appearing at 2073 cm^−1^ and 2000 cm^−1^. In contrast, the classic IR spectrum of polyacrylonitrile shows only the absorption peak at 2241 cm^−1^ [40]. It is proposed that the appearance of these lower frequency peaks, attributed to the reduction in C≡N bond strength, results from charge induction. As illustrated in Figure 4a, the electron lone pair of the glycidyl ether enhances the dipole moment of the nitrile group, namely lowering the electron density of the triple bond. Additionally, the carbonyl group of the G units shows a high-frequency C=O stretching peak at 1784 cm^−1^. This shift can be attributed to charge transfer from the oxygen to the π bond due to dipole repulsion (Figure 4b), resulting in increased bond strength of the double bond. The intensity of this peak rises as the *x/y* ratio increases. This phenomenon requires a highly uniform distribution of G units within the hydrophobic A_x_G_y_ block, ensuring that the close proximity of the nitrile and glycidyl carbonyl groups in the dry state creates microenvironments that facilitate such charge movements. In summary, it is unlikely that the G units form short segments within the hydrophobic block. (3) The S_z_ block displays its characteristic sulfonate features: the asymmetric stretching of the S=O bond displays at 1308 cm^−1^, which is slightly higher than that of an aromatic sulfonate [41] due to the absence of π-π conjugation. Additionally, the symmetric S=O and the S-O-K^+^ stretching bands are observed at 1044 cm^−1^ and 964 cm^−1^, respectively. Furthermore, as expected, the carbonyl stretching absorption peak of the S unit appears at 1735 cm^−1^, and its intensity increases with the increase in the length of the A_x_G_y_ block.

**Figure 5:**
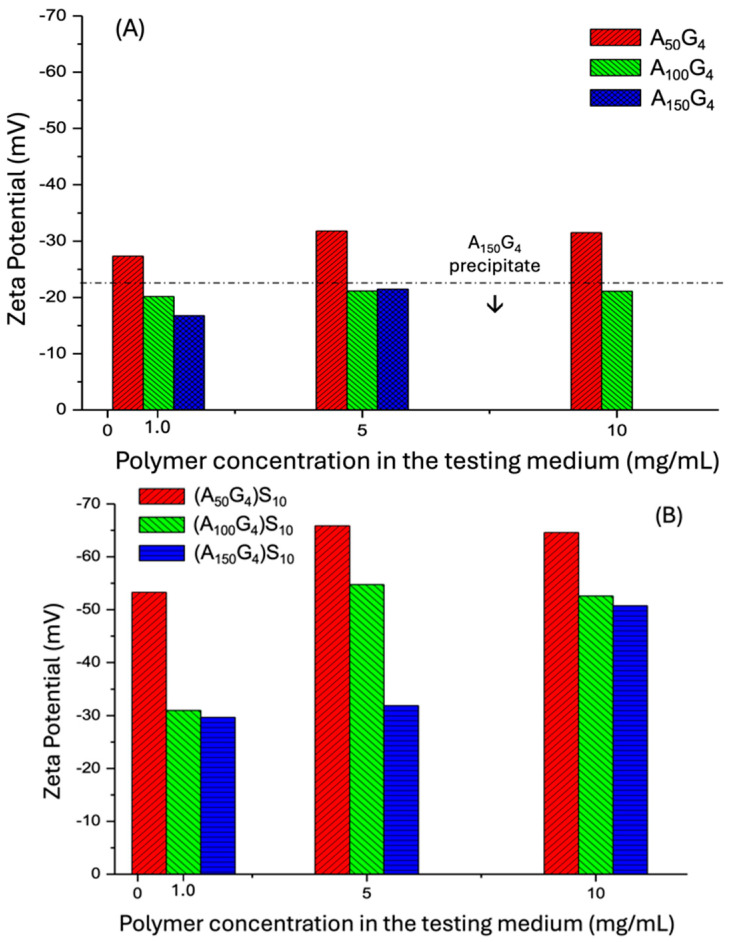
Zeta potentials of the colloidal dispersions of various copolymers in water within the designated concentration range: (**A**). The dispersed colloidal particles are formed of the hydrophobic copolymers A_x_G_y_, in which the dash line intercept with ζ = −22 mV represents the zeta potential of pure polyacrylonitrile in water at pH = 3 quoted as a reference value. (**B**). The dispersed colloidal particles of the DIs in water. Each sample was measured three times consecutively, with no observed variation.

As described in Section 4.3.3, the zeta potential measurement requires meticulous preparation of the copolymer colloidal dispersion formed when water is introduced into the DMSO polymer solution—to ensure that the polymer colloidal particles formed of aggregated polymer chains, remain in a discrete and dispersed state in the final step, while the DMSO is removed through dialysis. This state is referred to as a binodal state, relying on kinetic stabilization. In this assessment, the six synthesized polymers were determined in pure aqueous medium where pH is close to 7 in Figure 5A. The preset concentrations of 1.0, 5, and 10 mg/mL, which are within the concentration boundary of the typical dilute polymer solution (<1 wt.%). It has been found that pristine polyacrylonitrile in water exhibits a ζ potential of −22 mV at pH 3 and −35 mV at pH 7 [42]. This behavior can be attributed to the formation of surface species, -C≡N⋅⋅⋅H^+^ OH^−^, where hydroxyl anions are prevalent in the diffuse plane of the electrical double layer, contributing to the negative zeta potential. In our measurements, the zeta potential decreases as the x/y ratio increases at the lowest concentration point: ζ_A50G4_ −27.5 mV < ζ_A100G4_ −20.0 mV < ζ_A150G4_ −16.3 mV. This observation suggests two effects: first, the presence of the monomer unit G decreases the negative surface charge level compared to the above reference at pH = 7, this is presumably due to the steric perturbation effect imposed by the G units on the surface double electric layer; second, the increase in the segment length of A leads to forming looser chain aggregates because of the conformational restriction effect, resulting in a relatively lower interfacial density of nitrile groups.

At the middle concentration point, samples A_100_G_4_ and A_150_G_4_ exhibit nearly identical ζ values of approximately −21 mV, which are slightly more negative compared to their values at the lowest concentration. The observation indicates that the rise in concentration promotes both the interfacial contact of the aggregates due to an increase in particle number and chain packing within them for these two copolymers. The former favors assisting the perturbation role of G, whereas the latter favors acquiring a negative charge; both arise from the higher x/y ratios. The resulting ζ values reflect a balance between these two opposing effects. In contrast, sample A_50_G_4_, with the highest G unit mole fraction (or the lowest x/y) that minimizes steric (conformational) hindrance to chain packing, exhibits a greater increase in surface charge density. On top of this, the least hydrophobic A_50_G_4_ among the three copolymers allows it to gain a higher interfacial area. Both lead to a more negative ζ value of −32.2 mV compared to its value at the lowest concentration. Furthermore, the most hydrophobic A_150_G_4_ is unable to maintain its colloidal dispersion in the continuous medium before reaching the highest concentration point. Meanwhile, the colloidal particles of A_100_G_4_ retain very close ζ values at the three dispersions, suggesting that coalescence of the copolymer chains counterbalances the increase in the number of aggregates. Furthermore, A_150_G_4_ exhibits a more negative ζ value at the middle concentration vs. at the low concentration. This phenomenon is attributed to a tradeoff between the larger conformational spatial demand of the A_150_G_4_ chains and their high hydrophobicity that limits an expansion of polymer–water interface. In consequence, the former factor assists with achieving a higher surface charge level due to the exposure of nitrile groups to the dispersion medium.

In Figure 5B, the zeta potentials of the three DI samples in the pure water medium show a shift toward more negative values, which can be attributed to the presence of anionic S blocks attached to the hydrophobic block, as expected. The DI molecules spontaneously form micelle structures in water, with the hydrophobic blocks associating inward and the outspreading S blocks. The term “colloidal particles”, as used below, encompasses both micelles and their aggregates. This behavior distinguishes them from the aggregation of the A_x_G_y_ copolymer chains. The following trends are observed: (i) dispersions with the lowest concentration exhibit less negative ζ values among the three concentrations studied, due to fewer solutes and hence S blocks; additionally, (A_100_G_4_)S_10_ shows slightly more negative ζ than (A_150_G_4_)S_10_ at this concentration. This suggests a marginally higher number of S blocks present in the diffuse plane of (A_100_G_4_)S_10_ particles per surface area. It is the result of a larger mean aggregation number that causes a lower interfacial area. For the same reason, (A_50_G_4_)S_10_ has noticeably shorter A segments between G units compared to the other two samples, resulting in the most compact micelle structure and, hence, a denser negatively charged surface. (ii) The same ζ magnitude pattern is observed at the middle concentration, with (A_100_G_4_)S_10_ showing an even more negative ζ than (A_150_G_4_)S_10_. This larger difference can be attributed to an increase in the disparity in chain mobility of the hydrophobic block between these two samples with the rise in concentration. (iii) As the concentration increases from the middle to the highest level, the surface charge of (A_150_G_4_)S_10_ colloidal particles increases noticeably, while that of (A_100_G_4_)S_10_ particles slightly decreases. Since less conformationally hindered chain conformation of (A_100_G_4_)S_10_ allows the copolymer chains to achieve a more compact assembly than (A_150_G_4_)S_10_, it results in a greater degree of aggregation and, consequently, larger colloidal particle sizes, which explains the observed decrease in surface area and ζ potential accordingly [43]. Regarding the effect of increased concentration on the zeta potential of (A_150_G_4_)S_10_, it is assumed that the more flexible chain conformation forms result in coils with larger spatial occupancy prior to DMSO removal by dialysis. As a result, colloidal particles with loose structures and a greater number are ultimately formed in pure water compared to those with shorter A-segments between G units. Both the movable colloidal particle structure and, more significantly, their higher surface area comprising hydrated S blocks contribute to a more negatively charged level. This pattern is also observed in the change in ζ for A_150_G_4_ from the lowest to the middle concentration (Figure 5A), which is, although, less pronounced in the absence of S-block hydration effects. (iv) The conformational effect-driven micelle aggregation phenomenon is also observed in the (A_50_G_4_)S_10_ dispersion at the highest concentration, where the ζ potential becomes less negative compared to its value at the middle concentration. This phenomenon arises from concentration-induced assembly, leading to the formation of larger micelles and, consequently, larger colloidal particles, which reduces the interfacial contact.

### 2.3. Analysis of the Spinodal Decomposition Behaviors of DIs in a DMSO-Water Medium

This section uses the Static Light Scattering (SLS) method to examine how the coil structures, owing to full solvation, of the two copolymer groups, A_x_G_y_ and (A_x_G_y_)S_z_, respond to the rise in water content in DMSO. As discussed earlier, these six structures are soluble in DMSO, primarily stabilized by dipole–dipole interactions between the -C≡N group of monomer unit A and the >S=O group of DMSO. Adding water to a DMSO solution of any of these polymers causes contraction of the polymer chains, with the extent of contraction depending on both the hydrophobic content, i.e., x/y ratio, and the H_2_O content (vol.%), as defined in Section 4.3.4, in the dispersion medium. Notably, none of these polymers can form a stable colloidal dispersion in pure water if the two-step transition protocol from a DMSO solution to a DMSO-water dispersion is replaced by directly vortex-mixing the copolymers with water. Previous studies have investigated the micellization of an amphiphilic diblock copolymer to form microparticles when water is introduced into its aprotic solution. These free colloidal particles undergo clotting, forming aggregates once the water contents reach certain thresholds [44,45].

**Figure 6:**
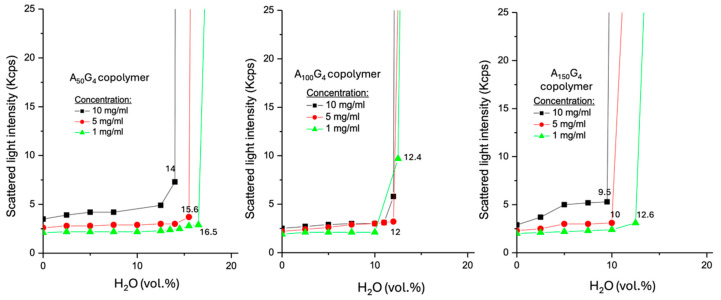
Scattered light intensities as a function of H_2_O (vol%) in the resulting A_x_G_y_ colloidal dispersions. The three concentrations listed in each Figure represent the initial DMSO solutions of the polymers investigated. Each sample was measured three times consecutively, with no observed variation.

Unlike these two studies that examined the polystyrene-polyacrylic acid (PS-*b*-PAA) diblock chain structure, the hydrophobic block, A_x_G_y_, in the present study reveals a charged polymer-water interface, as indicated by ζ potential measurements, a characteristic that the PS block lacks. Consequently, it is assumed that (A_x_G_y_)S_z_ DIs must have relatively higher HLB values, though they have not yet been quantitatively determined. In Figure 6, the SLS tests of the dispersions of the hydrophobic copolymers A_x_G_y_ support this awareness because they do not behave like PS. The scattered light intensity (SLI) of the pure DMSO solution at 0% water content (starting point) reflects the presence of the fully solvated polymer coils, which causes the SLI readings to weakly follow the expected concentration-based order. The SLI plots then gradually increase to varying extents, depending on the mole fraction G and concentration, as water is introduced into the three different dispersion systems.

When the water content approaches the critical water content (CWC), aggregation of the copolymer colloids begins. Accordingly, the final point on each SLI plot represents the critical state, marking the transition from the binodal to the spinodal region. The water contents of the dispersion media at these points are identified as CWCs, which are labeled in each plot. The sharp jump in SLI immediately after the CWC is attributed to the rapid precipitation of colloidal aggregates. It is important to emphasize that the pure water-based colloidal dispersions prepared for the zeta potential measurements (Section 4.3.3) are metastable, meaning they are pseudo-homogeneous and differ from those reaching CWC in the SLS experiment (Section 4.3.4). This examination observes the entire process starting, from the DMSO solution to the occurrence of massive phase separation. Figure 6 reveals the following trends:

(i) For a copolymer A_x_G_y_, an increase in concentration results in a lower CWC. This reflects the number-driven aggregation behavior.
(ii) At the low concentration, A_100_G_4_ shows the highest SLI (ca. 9.9 Kcps) at its CWC compared to the other two copolymers, whose SLI values are comparable. This is attributed to the balance between the ease of chain tangling and the realized interfacial area of the colloidal particles, which both contribute to SLI to different extents. This result aligns with their moderately negative ζ potential, as previously observed. At the middle concentration, all three copolymers exhibit comparable SLI at their CWCs due to a tradeoff between the surface of the colloidal particles and their surface characteristic. For example, A_150_G_4_ colloidal particles exhibit the lowest CWC, indicating the strongest occurrence of hydrophobic coagulation of many small colloidal particle sizes, and such a light aggregation assisted by the DMSO component should maintain a relatively higher portion of the initial interfacial area, which favors SLI [46]. Similarly, the higher interfacial exposure allows more nitrile groups to contribute to the surface charge as well.
(iii) At the highest concentration, A_50_G_4_ exhibits the highest SLI at its CWC among the three copolymers under identical conditions. This can be attributed to the impact of the highest colloidal particle surface associated with the maximum CWC (14%), as well as a more efficient surface chain packing extent facilitated by the reduced steric hindrance.
(iv) A_100_G_4_ exhibits concentration-independent CWCs, which can be attributed to the balance between competing factors: raising concentration promotes chain aggregation, while steric hindrance counteracts this effect. In contrast, both A_50_G_4_ and A_150_G_4_ show more pronounced variations in CWCs across different concentrations due to shifts in this balance as well as the leverage of their obviously diverse hydrophobic traits.

**Figure 7:**
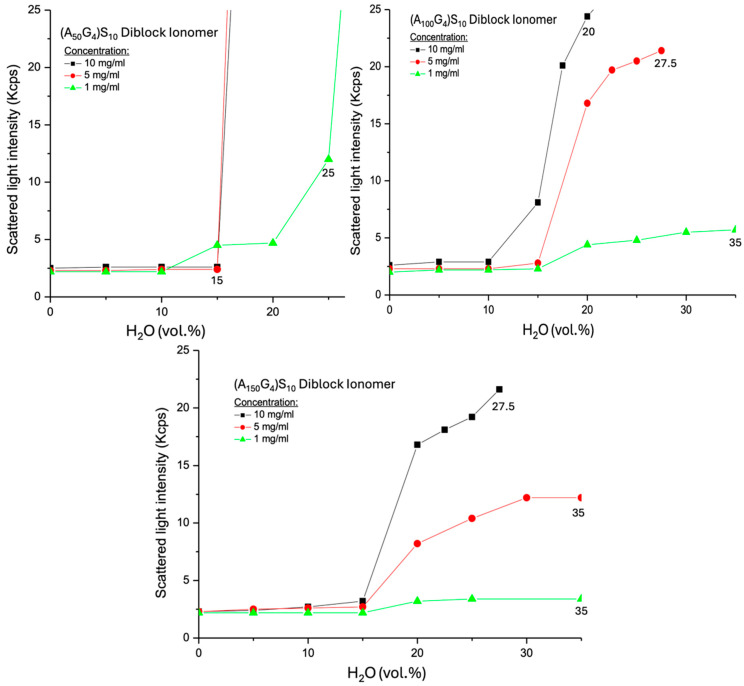
Scattered light intensities as a function of H_2_O volume fraction in the resulting (A_x_G_y_)S_z_ colloidal dispersions. The three concentrations in each Figure represent the initial DMSO solutions of the DIs before water was introduced into the solution. Each sample was measured three times consecutively, with no observed variation.

**Figure 8:**
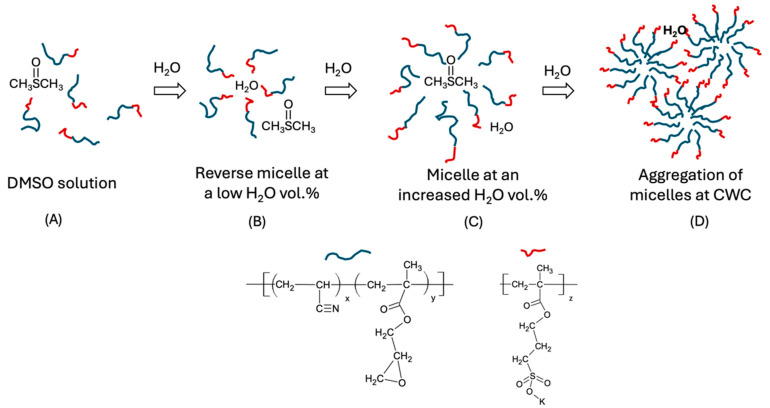
Conceptual illustration of the movement of DI chains with the continuous introduction of water into the DMSO-water dispersion medium. The blue and red curve segments represent the polymer blocks underneath, respectively. (**A**) DI molecules in DMSO, (**B**) Dilute water–induced assembly, (**C**) Formation of normal micelles at a high–water content, and (**D**) Water–driven micelle aggregation.

Now, regarding the three DIs, the DMSO solutions (H_2_O vol% = 0) exhibit nearly identical SLI values, regardless of chain composition and concentration (Figure 7). This phenomenon reflects the bulk liquid structure of the dilute polymer solution, where individual polymer chains move freely, surrounded solely by DMSO (Figure 8A) [47]. When water is added to the DMSO solution of (A_50_G_4_)S_10_, dispersions formed with the two higher concentrations (represented by the *red* (*r*) and *black* (*b*) lines in Figure 7) exhibit identical CWCs, with almost no change in SLI before reaching CWC. Furthermore, their CWCs are noticeably lower than that of the same type of dispersion with the lowest concentration (the *green* line (*g*)). This happening is deemed to occur in two stages after water is added: initially, the formation of reverse micelle upon the addition of water [47], during which highly solvated hydrophobic blocks (as shown in 8B) scatter light ineffectively. Then, with increasing water content, the inversion of the micelle likely takes place, resulting in the formation of normal micelles (8C) [48]. The high molar content of G in hydrophobic block (A_50_G_4_) promotes rapid micellization once the concentration in the DMSO-H_2_O medium exceeds the critical micelle concentration (CMC). CMC will also decrease as the water content increases. The *r* and *b* lines show identical CWCs and SLIs, which can be attributed to two factors: first, stronger water acceptance by reverse micelles (8B) with the two higher concentration cases, where the highly solvated A_50_G_4_ blocks in DMSO have minimal impact on light scattering; second, as the water content increases, the reverse micelles undergo swift micelle inversion (8C) and coalescence, occurring almost simultaneously in both dispersions (8D). In contrast, at the lowest concentration, represented by line *g* in Figure 7, (A_50_G_4_)S_10_, undergoes micelle inversion within the range from 10 to 15 vol.% water content, as indicated by the change in the slope of the SLI. This is followed by micelle contraction, causing the SLI to increase until micelle aggregation begins at 25 vol.% water content (8D). At this CWC, the SLI is significantly higher compared to that of the same DI at the two higher concentration cases. This is likely because the latter two dispersions contain larger colloidal particles, formed by the aggregation of more micelles. The resulting larger particles have reduced surface areas, resulting in less intense scattering of incident light. It is likely that these larger particles are more susceptible to the increase in water content, ending up with earlier phase separation, viz., showing the lower and identical CWCs.

Furthermore, both (A_100_G_4_)S_10_ and (A_150_G_4_)S_10_ DI molecules unveil similar light scattering patterns to that exhibited by (A_50_G_4_)S_10_ at the lowest concentration, namely, micelle formation via two stages. However, there are three obvious different phenomena associated with their chain structures:

- The higher concentration leads to greater SLI following the formation of normal micelles.
- (A_150_G_4_)S_10_ micelles exhibit weaker SLI than (A_100_G_4_)S_10_ micelles at equivalent water content.
- No observable precipitation occurs in the dispersions of these two DIs.

The first phenomenon arises from the DMSO-swollen micelle interior, in which an increase in chain conformational flexibility due to longer A segments between G units, as described above, discourages steric packing of the hydrophobic block. Therefore, the increase in concentration leads to the formation of more micelles and, subsequently, more colloidal particles. The second phenomenon relates to the looser micelles and the resulting colloidal particle structures composed of the (A_150_G_4_)S_10_ chains, similar to the influence of particle structure on the ζ potential magnitude discussed earlier. The third phenomenon arises from the partially solvated micelle interior, where the motion of hydrophobic blocks promotes a loosely packed hydrophilic exterior. This increases the buoyancy of micelles in the liquid medium and reduces their tendency to coalesce, with charge repulsion serving as an additional contributing factor [49].

The selected SLS testing results for these three DI samples are compared with those of two similar aqueous colloidal dispersions in Table 1. These reference dispersions consist of colloidal particles made from amphiphilic diblock copolymers: PEG54-P(AA/VE6/γTCP29)140 [50] and PEO4000-b-PB1800 [51], where EG is ethylene glycol, AA is acrylic acid, VE is vinyl ether, γTCP is γ-tocopherol, EO is ethylene oxide, and B is butylene. The former has a calculated molecular weight (Mw) of 1,248,047 Da, while the latter has an Mw of 273,200 Da. It is evident that the significantly higher Mw of these two reference systems, compared to the DI samples (as discussed in Section 2.1), contributes to a greater scattered light intensity. Furthermore, the hydrophilic PEG blocks can form a more compact hydrogel layer around the colloidal particles due to their linear chain structure.

### 2.4. Determination of Copolymer Colloidal Particle Size Distribution in Water Using DLS

**Figure 9:**
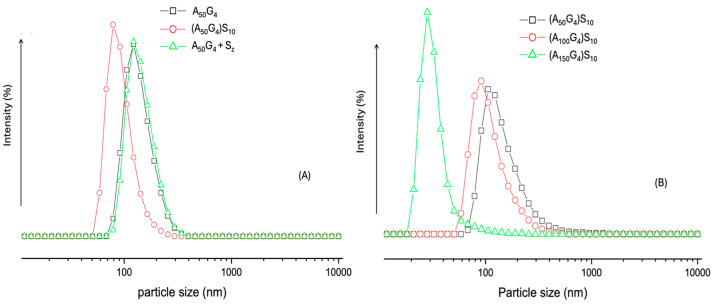
The DLS particle size distribution analysis of the aqueous dispersion of the six copolymers (10 mg/mL). (**A**) Exhibits the particle size distributions of copolymer A_50_G_4_, diblock copolymer (A_50_G_4_)S_10_, and a mixture of A_50_G_4_ and Sz; (**B**) exhibits the particle size distributions of the three diblock copolymers.

The water-based colloidal dispersions used for this measurement are the same as those used to determine ζ potential. Therefore, the particle size distributions of a sample should correlate with its ζ potential variation. As previously mentioned, there are two types of colloidal particles: coagulates of A_x_G_y_ copolymers and of the (A_x_G_y_)S_z_ micelles as well as coagulates. For the former type of colloidal particles, A_50_G_4_, was selected for analysis because this copolymer exhibits significant chain coalescence even in highly diluted aqueous environments (e.g., 1 mg/mL), as indicated by ζ-potential measurements. Among the three synthesized copolymers, A_50_G_4_ experiences the least conformational hindrance during chain entanglement due to its highest mole fraction of G units in the copolymer chains. The colloidal particles of the A_50_G_4_ copolymer in water present a unimodal size distribution curve centering at ca. 120 nm and a range from 70 to 140 nm (Figure 9A). This evidences that each particle comprises numerous A_50_G_4_ chain coils as their radius of gyrations (about 1.2 nm [52]) is far smaller than the particle sizes within the above range. Correspondingly, these particles exhibit surface charges (ζ = −31 mV) at the same concentration, providing charge stabilization for colloidal dispersion. In contrast, the colloidal particles formed of (A_50_G_4_)S_10_, show the most abundant particle size at 80 nm with a distribution range from 50 to 120 nm. This indicates that the colloidal particles of (A_50_G_4_)S_10_ achieve a greater surface area compared to that of the colloidal particles formed of A_50_G_4_. The presence of a negatively charged layer (−65 mV) consisting of −S_10_ anionic blocks on each micelle due to the self-assembling effect [34] offers the role to stabilize smaller colloidal particles (Figure 8D). Furthermore, when equal concentrations (10 mg/mL) of the copolymer A_50_G_4_ and an anionic oligomer, S_z_, are blended together, the resulting particle sizes and their distribution still remain unchanged with respect to the pristine A_50_G_4_. The fact that the highly water-soluble S_z_ chains (refer to Section 4.2) are untied to the A_50_G_4_ aggregates causes this phenomenon. It should be noted that random association of polymer coils happens in the A_50_G_4_ aggregates but rather in the interior of individual (A_50_G_4_)S_10_ micelles.

**Figure 10:**
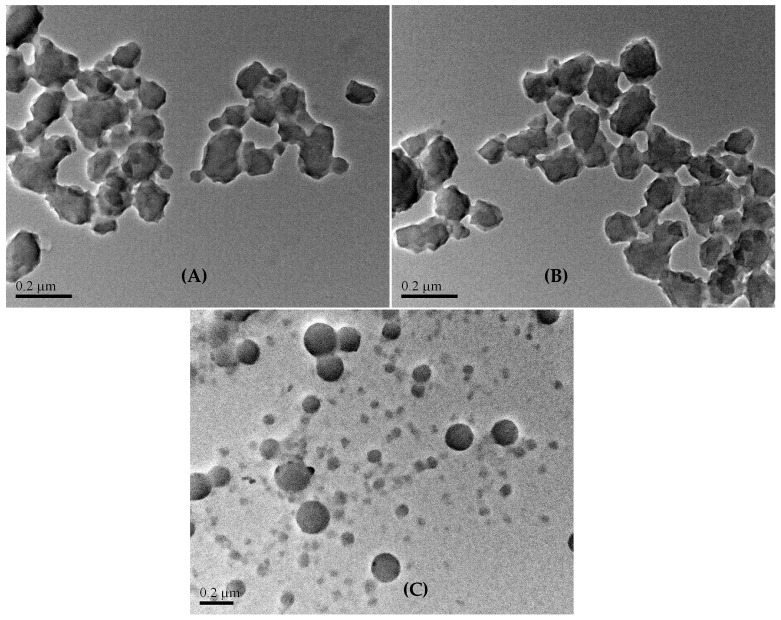
TEM images of the colloidal particles formed of the following: (**A**) A_50_G_4_ copolymer; (**B**) A_50_G_4_ copolymer and S_z_ anionic oligomer; and (**C**) (A_50_G_4_)S_10_ ionomer in water medium.

The TEM images of the above three kinds of colloidal particles can be divided into two groups, (A) and (B) vs. (C), according to particle size and shape (Figure 10). The former group exhibits irregular, rough, and interconnected particles, which are the characteristic of the aggregation of random polymer coils into individual particles. These particles connect via “necks” together during the drying course (refer to Section 4.3.5). In addition, the joining formation of colloidal particles by A_50_G_4_ and anionic oligomer S_z_ does not noticeably alter particle shape and size, which aligns with what has been concluded by the DLS measurement. Meanwhile, the mean particle sizes and the related standard deviations of these three samples, as evaluated from the TEM images, are 98.5 ± 42.9 nm for A_50_G_4_, 118.2 ± 48.7 nm for S_z_/A_50_G_4_, and 62 ± 42 nm for (A_50_G_4_)S_10_. To support these statistical values, charts (Figures S1–S3) illustrating the number of particles counted vs. particle size ranges are provided in the Supporting Information Section. Notably, image (C) exhibits spherical and discrete particles, primarily ranging from 20 to 108 nm, corresponding to micelles assembled by (A_50_G_4_)S_10_ molecules. The distribution of the image-shown particle sizes closely aligns with the DLS results as described above. Briefly, the chain aggregates of A_50_G_4_ and the hydrophobic cores constructed by the (A_50_G_4_) blocks have very different chain-associated structures.

Regarding the particle size distribution curves of the (A_100_G_4_)S_10_ and (A_150_G_4_)S_10_ dispersions (Figure 9B), they display smaller particle sizes, especially the latter one that has the most abundant particles with sizes of 30 nm, but that of the former one is 90 nm. This diversity can be attributed to the greater conformational restrictions among the A_150_G_4_ blocks compared to those within the A_100_G_4_ blocks, as previously described. In consequence, micelles formed by (A_150_G_4_)S_10_ exhibit lower chain assembling numbers, resulting in smaller and less compact micelle structures. The colloidal particles formed by the coalescence of micelles, as illustrated in Figure 8D, in these two dispersions, therefore, exhibit different size distribution profiles. It was also noticed that these two dispersions gradually became more obvious translucent after about 24 h, implying their colloidal particles undergo continuous coagulation because of the relatively weaker surface charge stabilization effect (Figure 5B). On the contrary, the (A_50_G_4_)S_10_ dispersion showed no sign of any noticeable phase separation even after a month of storage despite the largest particle sizes among the three dispersions. The highest surface charge (−65 mV) on (A_50_G_4_)S_10_ colloidal particles ensures their stability against further coagulation.

**Figure 11:**
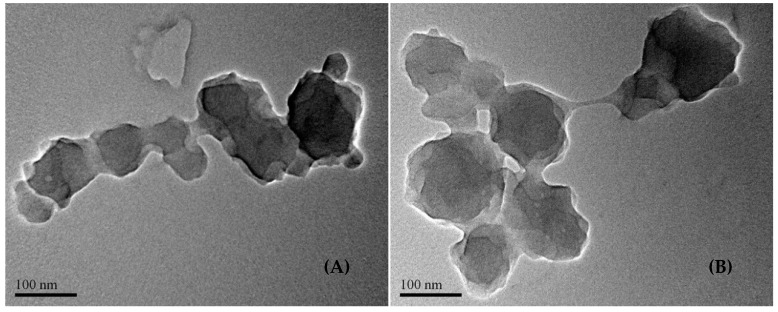
TEM images of the aggregates formed of the diblock (A_100_G_4_)S_10_ ionomer (**A**) and of the diblock (A_150_G_4_)S_10_ ionomer (**B**), respectively.

The aggregation propensity of (A_100_G_4_)S_10_ and (A_150_G_4_)S_10_ DIs, while their dispersions were dried, was verified by TEM, as shown in Figure 11. Both present irregular particles and necking among them. The former contains particles approximately 50 to 130 nm in size, closely aligning with their DLS measurements. However, the particles lose their spherical shape and tend to stick together, likely due to the fusion of micelles surrounding the colloidal particles during drying. This fusion contributes to the formation of necks between particles. This tendency is more pronounced when the colloidal particles of (A_150_G_4_)S_10_ coagulate, attributed to their structural characteristics: smaller particles composed of the loose micelles in the dispersion, as previously discussed. The coagulates also exhibit a loose bulk structure, as evidenced by their relatively translucent appearance in Figure 11B.

## 3. Conclusions

This study explores two types of physically associated microgel structures in the form of colloidal particles derived from the copolymers A_x_G_y_ and (A_x_G_y_)S_z_. A_x_G_y_ is a random copolymer, while (A_x_G_y_)S_z_ is a diblock ionomer (DI) comprising a hydrophobic (A_x_G_y_) block and a hydrophilic anionic S_z_ block. The subscripts represent the stoichiometric ratios of the polymerization feedstock. Both types of copolymers were synthesized via ATRP, maintaining a constant molar ratio of monomers to catalyst despite variations in the dosage of A (x). Consequently, the copolymers exhibit similar average molecular weights, approximately 36,000 Da for A_x_G_y_ and 63,000 Da for (A_x_G_y_)S_z_. This study focuses on the impact of the mole fraction of the glycidyl methacrylate unit (G) within the acrylonitrile unit (A) dominant copolymer chain or block on the properties of colloidal particles formed in a DMSO–water medium. The following conclusions were drawn from this investigation.

1. Both types of copolymers dissolve in DMSO as solvated coils and transition to colloidal particles upon water incorporation. In the case of A_x_G_y_, the colloidal particles form as random aggregates of copolymer chains, whereas for (A_x_G_y_)S_z_, they assemble into micelles, which subsequently coagulate to form colloidal particles. The resulting colloidal dispersions remain in a metastable state when DMSO is dialyzed from the medium.
2. The colloidal particles of A_x_G_y_ dispersed in water exhibit a weak negative ζ potential, which increases with a higher mole fraction of the amphiphilic unit G (viz. a higher y/x), favoring the generation of surface charge. Reciprocally, a higher x/y ratio enhances the ζ potential by promoting looser chain packing and, hence, surface area due to increased conformational flexibility. A similar trend is observed in aqueous dispersions of (A_x_G_y_)S_z_, though these exhibit significantly more negative ζ potentials.
3. Scattered light intensity (SLI) analysis reveals the coagulation process of A_x_G_y_ chains as water content increases in the DMSO-H_2_O dispersion medium. Clear spinodal decomposition (phase separation) occurs at critical water contents, generally following the trend that a higher x/y ratio corresponds to a lower critical water content, though this is slightly influenced by chain conformation. In contrast, (A_x_G_y_)S_z_ exhibits no distinct phase separation thresholds due to the hydrated exterior, except for the dispersion with the smallest x/y ratio, where compact micelles form, which triggers sedimentation.
4. Dynamic light scattering analysis reveals the colloidal particle size distribution of the three (A_x_G_y_)S_z_ in water, showing that the largest x/y ratio produces the smallest particles, while the smallest x/y ratio results in the largest particles. The hydrophobic chain conformational flexibility plays a key role in leveraging the colloidal particle sizes. Notably, the colloidal particles with the largest sizes are highly stable, leaving behind nano-spherical structures (ca. 20–200 nm) after drying. The nanospheres function effectively as microgels, featuring a hydrophilic shell and a hydrophobic core.

Lastly, to explore the effects of doping the hydrophobic block on colloidal stability and particle morphology, future studies will prioritize three key ideas: i. conducting a ^1^H-NMR study of the micelles formed by the three DI samples to gather information about the dynamic state of the hydrophobic block within the micelles; ii. incorporating a fluorescent probe within the interior of the micelle to examine how the conformational flexibility of the chains affects the emitted light frequency; iii. substituting the acrylonitrile segment with a soft and bulky segment, such as 2-*tert*-butyl-1,3-butadiene, to investigate the impact of increased chain bulkiness and reduced chain cohesion on the behavior of microgel particles.

## 4. Materials and Methods

### 4.1. Chemicals

3-Sulfopropyl methacrylate potassium salt (SPM, 98%, M = 246), acrylonitrile (AN, ≥ 99%, M = 53), glycidyl methacrylate (GMA, 97%, M = 142), copper(I) bromide (CuBr, 98%), 2,2′-bipyridyl (bpy, 98%), 2-bromopropionitrile (BPN, 98%), dimethylsulfoxide (DMSO, HPLC-grade), dimethylformamide (DMF) were supplied by Sigma-Aldrich Pte Ltd., Singapore and used without further purification. The inhibitors in AN and GMA monomers were removed by passing them through an alumina column from Sigma-Aldrich Pte Ltd. Singapore.

### 4.2. One-Pot Synthesis of the Block Copolymer (A_x_G_y_)S_z_ by Atom Transfer Radical Polymerization (ATRP)

The synthesis protocol was identical to that described in our previous work [35]. However, to clarify the following steps, it is important to note that ethylene carbonate (EC) was used as the solvent for the synthesis of (A_x_G_y_), in which the composition of the reactant feed has a molar ratio of [A]_0_/[G]_0_/[RX]_0_/[CuX]_0_/[bpy]_0_ = 100/4/1/0.1/0.3. For attaching the S_z_ block to (A_x_G_y_), monomer S was first dissolved in DMSO and then introduced into the reactor where (A_x_G_y_) was just synthesized, with the S_z_ block growing on (A_x_G_y_) in the binary solvent mixture of EC/DMSO. The volume ratio of EC to DMSO is approximately one. Finally, the DI, (A_x_G_y_)S_z_, was separated by adding an excess of a diethyl ether/methanol mixture (40/60) into the polymerization solution. The ionomer was then filtered and vacuum-dried at 40 °C for 24 h. In this study, three types of DI samples were synthesized by varying the stoichiometric coefficient x in the formula (A_x_G_y_)S_z_ from 50 to 150, whereas, keeping the coefficients y = 4 and z = 10 fixed for all three DIs. The six copolymers, three A_x_G_5_ and three (A_x_G_4_)S_10_, were synthesized using the same molar ratio of monomers to catalyst as stated above. Moreover, for comparison purposes, an anionic oligomer, S_z_, was prepared using a similar molar ratio [S]_0_/[RX]_0_/[CuX]_0_/[bpy]_0_ = 10/0.1/0.01/0.03 to formulate the feed.

### 4.3. Characterization Methods

The following five sections describe five different instrumental methods used to characterize the hydrogel microparticles, both in their dispersion medium of water and DMSO, as well as in their dry form.

#### 4.3.1. Chromatography Analysis

- The time-dependent conversion of the two monomers, AN and GMA, during ATRP to synthesize the hydrophobic block (A_x_G_y_) was monitored using gas chromatography (GC) analysis. This polymerization followed the method established by Matyjaszewski et al. [53]. Samples (1 mL) were withdrawn from the Schlenk flask at predetermined intervals (0, 0.5, 1, 3, 6, and 23 h) using a glass syringe. Each sample was immediately introduced in 2 mL THF to precipitate the polymer component and keep the monomers in THF. The resulting suspension was filtered through 0.45-μm Millipore Millex^®^ HN filtration paper, and the filtrate was analyzed using a Shimadzu GC-2010 equipment (Kyoto, Japan) equipped with a flame ionization detector, for which THF acted as the internal standard. The calibration standards were prepared by mixing a series of AN (0.0152–6.076 mmol), respectively, with 4 mole % GMA in 2 mL THF in sample vials. After the same filtration purification as described above, the clear solution in each vial was injected into the GC to establish the calibration curve. Quantitatively, the monomer conversion was the mean of the conversions of the two monomers.

Calibration standards were prepared by mixing a series of AN (0.0152–6.076 mmol) with 4 mol % GMA in 2 mL of THF in sample vials. After undergoing the same filtration purification as described above, the clear solution in each vial was injected into the GC to establish the calibration curve. Monomer conversion was quantified as the average of the conversions of the two monomers.

b. Determination of the molecular weights, ${\bar{M}}_{w}$ and ${\bar{M}}_{n}$, of copolymers A_x_G_y_ by the gel permeation chromatography (GPC). The molecular weights of the copolymers A_x_G_y_ were determined using the gel permeation chromatography (GPC) method. The measurements were carried out on a Waters GPC system (Milford, MA, USA) equipped with Waters Styragel columns, a Waters—2487 dual wavelength UV detector, and a Waters—2414 refractive index detector. DMF was used as the eluent at a flow rate of 1.0 mL/min.

Copolymer samples (from Section 4.2) were collected at various polymerization times (0 to 21 h) and redissolved in DMF to prepare 1 mg/mL solutions. These solutions were filtered through 0.45-μm Millipore Millex^®^ HN filtration membranes and stored in 1 mL sample vials for GPC analysis.

Monodispersed polystyrene (PS) standards with molecular weights of ${\bar{M}}_{w}\;$= 2970, 13,700, 55,000, 197,000, and 1,370,000 Da were used to generate calibration curves for molar mass determination based on the retention time in GPC.

#### 4.3.2. Infrared Spectroscopy Analysis of the Copolymers of A_x_G_y_ and (A_x_G_y_)S_z_

FT-IR spectra of the random copolymer A_x_G_y_ and the diblock ionomer (A_x_G_y_)S_z_ were recorded using a Shimadzu FTIR spectrometer (IRAffinity-1, Columbia, MD, USA) with a KBr pellet window. The pellet was prepared by grinding 0.1g of KBr powder with 1 mg of polymer in a mortar, followed by compressing the mixture in a pellet die at approximately 8000 psi to form a 10 mm round, thin pellet. The spectra were obtained from 40 scans over a wavenumber range of 400 to 4000 cm^−1^. The results confirm the presence of the desired organic functionalities.

#### 4.3.3. Zeta Potential Measurement of the Copolymers of A_x_G_y_ and (A_x_G_y_)S_z_

The zeta potentials of the synthesized copolymers in water-DMSO solutions were measured using a Zetasizer Nano ZS (Malvern, Worcestershire, UK). To prepare the samples for the test, deionized water (Milli-Q, Darmstadt, Germany) was incrementally added to a given volume of the pre-prepared DMSO solution of the copolymers (100 mg/mL). Water was added in 100 μL increments with shaking to ensure homogeneity until the target final concentrations of 1, 5, and 10 mg/mL in water were achieved after deducting the DMSO component. The resulting solutions were then dialyzed against deionized water for 24 h using a dialysis membrane to remove DMSO, following the procedure described in reference [54]. Finally, the samples were sealed and stored at ambient temperature for subsequent measurement.

#### 4.3.4. Static Light Scattering (SLS) and Dynamic Light Scattering (DLS) Measurements

SLS measurement was conducted to assess the effects of the chain structures of the two types of copolymers on the zeta potential, which reflects the interfacial physical state between the polymer and the dispersion liquid medium. The measurements were performed using a Goniometer system (Brookhaven 90+, Nashua, NH, USA) with incident light at a wavelength of 532 nm. Stock solutions of the six synthesized copolymers, A_x_G_y_ and (A_x_G_y_)S_z_, were prepared by dissolving the copolymer powders in DMSO at concentrations of 1, 5, and 10 mg/mL for each copolymer. Equal initial volumes (V_0_) of these solutions were then stepwise diluted with Milli-Q deionized water (V_w_) using a micro-syringe. Static light scattering (SLS) data were obtained by measuring the scattered light intensity (Kcps) as a function of V_w_/V_0_ (%) at a controlled temperature of 25.0 ± 0.1 °C.

Dynamic light scattering (DLS) measurements for particle size were conducted using a Malvern Zetasizer Nano ZS, the same instrument used for zeta potential analysis in Section 4.3.3, to characterize colloidal particles in water. The samples, consisting of pure aqueous solutions at a concentration of 10 mg/mL, were prepared following the protocol described in Section 4.3.3.

#### 4.3.5. Transmission Electron Microscopy (TEM)

The TEM examination used the same aqueous colloidal dispersions as those for conducting DLS particle size determination, but the concentration of particles was kept at 5 mg/mL for all the dispersions. The measurement protocol includes the addition of a drop of the dispersion onto a TEM sample grid, then the grid was exposed to air for drying for several minutes and finally placed in the vacuum chamber of the microscope (JEOL 2100, Tokyo, Japan) to start the examination.

## Figures and Tables

**Table 1:** Benchmarking the SLI readings of the selected dispersions of the three DIs.

Copolymer	Concentration (mg/mL)	Dispersion Medium	SLI (Kcps)
(A_50_G_4_)S_10_	8.7 ^a^	water	2.7
(A_100_G_4_)S_10_	8.7	-	5.8
(A_150_G_4_)S_10_	8.7	-	3.3
PEG_54_-P(AA/VE6/γTCP_29_)_140_	0.1	water	300
PEO_4000_-*b*-PB_1800_	1.18	water	180

^a^. The concentration is calculated using the SLI values of the dispersions at H_2_O (vol.%) = 15% as shown in Figure 7.

## Data Availability

PhD thesis “Acrylic, Aliphatic Ionomer, Polymer Electrolyte Membrane, Direct Alcohol Fuel Cell, Radical Polymerization, In situ Cross-linking” by David Julius (accessed on 25 May 2011). http://scholarbank.nus.edu.sg/handle/10635/30298.

## Supplementary Materials

The following supporting information can be downloaded at: https://www.mdpi.com/article/10.3390/gels11040231/s1, Figure S1: The particle size distribution of the dry A_50_G_4_ sample, based on its TEM image. The right vertical axis represents the cumulative percentage of particles as their size increases. The size of each particle is measure by its longest dimension; Figure S2: The particle size distribution of the dry S_z_/A_50_G_4_ sample, based on its TEM image. The right vertical axis represents the cumulative percentage of particles as their size increases. The size of each particle is measure by its longest dimension; Figure S3: The particle size distribution of the dry (A_50_G_4_)S_10_ sample, based on its TEM image. The right vertical axis represents the cumulative percentage of particles as their size increases.
