# Supplementary material for: Microgel with a Core—Shell Particulate Structure Formed via Spinodal Decomposition of a Diblock Ionomer Containing a Doped Hydrophobic Moiety
Source: Gels. 2025 Mar 22;11(4):231. doi: 10.3390/gels11040231 (PMC12027152; doi:10.3390/gels11040231)
Supplement: Supplementary file 1 [file gels-11-00231-s001.zip › gels-3389005-supplementary.pdf]

gels

MDPI

The Supplementary Materials

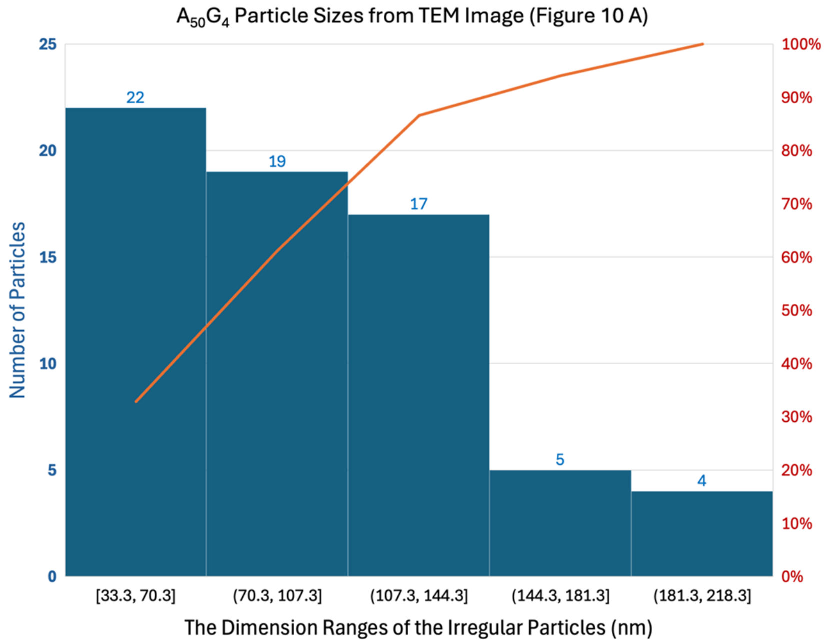

Figure S1. The particle size distribution of the dry $A_{50}G_4$ sample, based on its TEM image. The right vertical axis represents the cumulative percentage of particles as their size increases. The size of each particle is measure by its longest dimension.

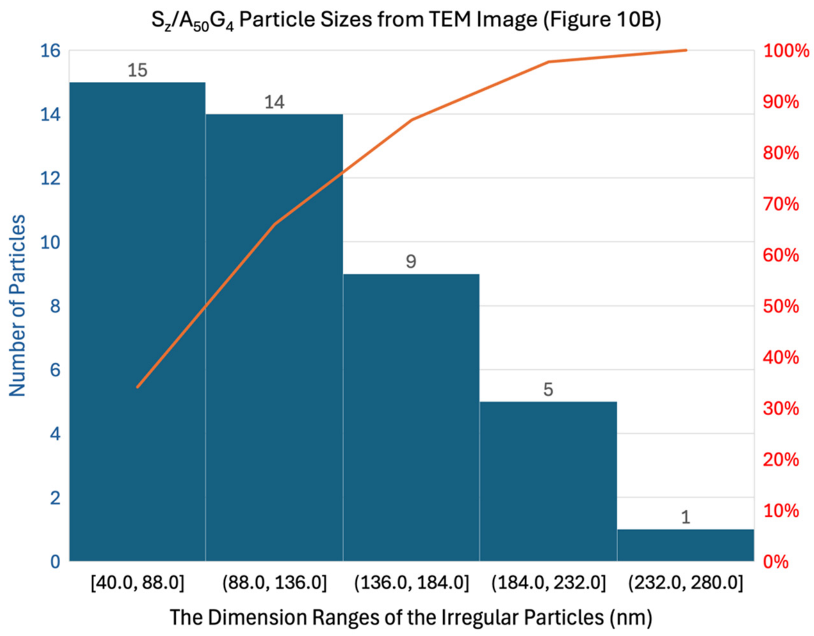

Figure S-2. The particle size distribution of the dry $S_z/A_{50}G_4$ sample, based on its TEM image. The right vertical axis represents the cumulative percentage of particles as their size increases. The size of each particle is measure by its longest dimension.

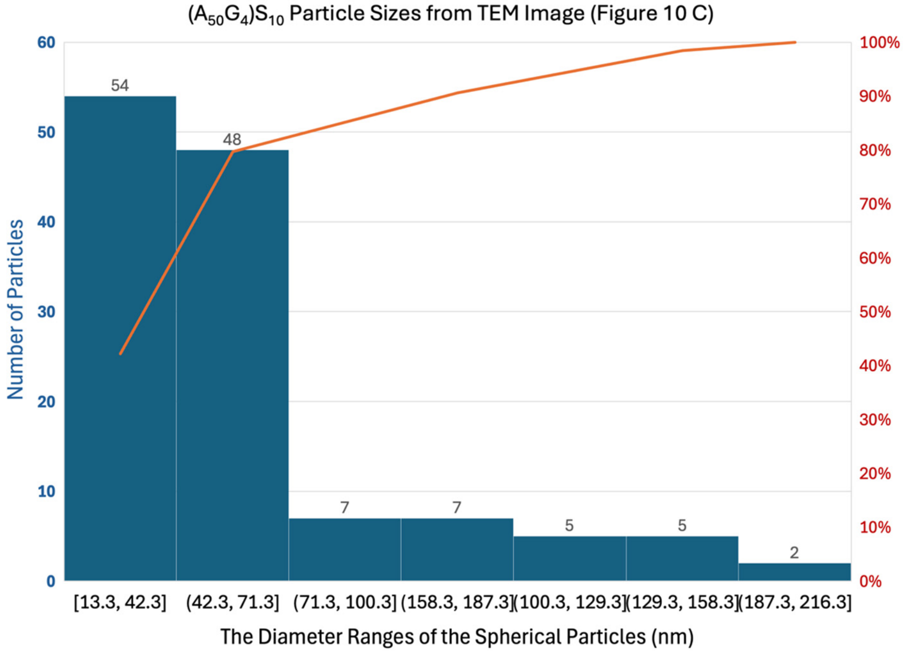

Figure S3. The particle size distribution of the dry $(A_{50}G_4)S_{10}$ sample, based on its TEM image. The right vertical axis represents the cumulative percentage of particles as their size increases.
